# Transient Overexpression of Sonic Hedgehog Alters the Architecture and Mechanical Properties of Trabecular Bone

**DOI:** 10.1359/jbmr.090313

**Published:** 2009-03-30

**Authors:** Maija Kiuru, Jason Solomon, Bassem Ghali, Marjolein van der Meulen, Ronald G Crystal, Chisa Hidaka

**Affiliations:** 1 Department of Genetic Medicine, Weill Medical College of Cornell University New York, New York, USA; 3 Tissue Engineering, Regeneration and Repair Program, The Hospital for Special Surgery New York, New York, USA; 4 Sibley School of Mechanical and Aerospace Engineering, Cornell University Ithaca, New York, USA

**Keywords:** osteoblasts, osteoclasts, hedgehog, bone remodeling, adenovirus

## Abstract

Bone formation and remodeling involve coordinated interactions between osteoblasts and osteoclasts through signaling networks involving a variety of molecular pathways. We hypothesized that overexpression of Sonic hedgehog (Shh), a morphogen with a crucial role in skeletal development, would stimulate osteoblastogenesis and bone formation in adult animals in vivo. Systemic administration of adenovirus expressing the N-terminal form of Shh into adult mice resulted in a primary increase in osteoblasts and their precursors. Surprisingly, however, this was associated with altered trabecular morphology, decreased bone volume, and decreased compressive strength in the vertebrae. Whereas no change was detected in the number of osteoclast precursors, bone marrow stromal cells from Shh-treated mice showed enhanced osteoclastogenic potential in vitro. These effects were mediated by the PTH/PTH-related protein (PTHrP) pathway as evidenced by increased sensitivity to PTH stimulation and upregulation of the PTH/PTHrP receptor (PPR). Together, these data show that Shh has stimulatory effects on osteoprogenitors and osteoblasts in adult animals in vivo, which results in bone remodeling and reduced bone strength because of a secondary increase in osteoclastogenesis.

## INTRODUCTION

The coordinated interaction of osteoblasts and osteoclasts is a requisite for normal bone modeling during skeletogenesis and remodeling throughout life.(1–3) Several molecular pathways regulate these interactions, including the hedgehog proteins and downstream activators such as bone morphogenetic protein (BMP), Wnt, PTH-related protein (PTHrP), and runt-related transcription factor 2 (Runx2).(1–3) Many of these mechanisms have been elucidated in a developmental context through the use of transgenic mice. However, studies of these pathways in adult bone could promote the translation of this knowledge to clinical applications, such as the treatment of osteoporosis or the enhancement of graft incorporation.

Hedgehog proteins, Sonic hedgehog (Shh), Indian hedgehog (Ihh), and Desert hedgehog (Dhh), are secreted morphogens that have crucial roles in embryonic patterning and the formation of a variety of organs including the skeleton.(4,5) For example, mice lacking Shh show defective development of vertebrae and limbs caused by abnormal patterning and endochondral ossification.(6) Activity of Shh induces osteoblast formation from mesenchymal stem cells through BMP(7) and PTHrP(8) pathways. In addition, Shh has been shown to be involved in early fracture repair and enhance bone defect repair in vivo.(9) The Ihh, secreted by osteoblasts and prehypertrophic chondrocytes of the growth plate, is a central regulator of endochondral bone growth through effects on both chondrocytes and osteoblasts.(10–15) Because signaling through the Patched1 (Ptc1) receptor is common to Ihh and Shh, their functions may overlap, and several studies have used them interchangeably to elucidate various roles in bone development.(4)

In the context of these previous studies, we hypothesized that Shh will stimulate osteoprogenitor cells and enhance bone formation in adult animals in vivo. Our strategy was to transiently overexpress Shh in mice using intravenous administration of adenovirus encoding a soluble N-terminal form of Shh.(16) Our results show that, surprisingly, overexpression of Shh alters trabecular morphology leading to diminished compressive strength of the bone. As expected, Shh treatment increases the number of osteoblasts, osteoblast progenitors, and mesenchymal stem cells. However, whereas the number of osteoclast precursors are not affected, the number of osteoclasts increases because of enhanced osteoclastogenic capacity of the marrow stroma. This enhanced osteoclastogenic activity is caused, at least in part, by upregulation of the PTH/PTHrP pathway.

## MATERIALS AND METHODS

### Adenovirus vectors

All adenovirus (Ad) vectors were serotype 5 and replication deficient with deletions in the E1a, E1b, and E3 regions of the Ad genome. The expression cassette of AdShhN contains the cytomegalovirus promoter/enhancer and encodes the N-terminal portion of murine Shh with a stop codon introduced to terminate translation immediately downstream of the glycine residue at position 198 of the Shh peptide, allowing it to be more diffusible than intact Shh.(16) A vector identical to AdShhN but lacking a cDNA coding sequence, AdNull, was used as a control. The vectors were propagated, purified, and characterized as previously described.(17,18)

### Experimental animals

Using an approved institutional animal committee protocol, C57BL/6 male mice 6–8 wk old (Jackson Laboratories, Bar Harbor, ME, USA or Taconic, Germantown, NY, USA) were administered AdShhN 5 × 10^10^ pu in 100 μl volume PBS, AdNull 5 × 10^10^ pu, or PBS alone as controls in the lateral tail vein and were killed 18 or 30 days later. Levels of murine Shh were quantified using a mouse Shh ELISA kit (R&D Systems, Minneapolis, MN, USA) in serum collected by cardiac puncture at death (day 18).

### Microscopic morphology

Isolated lumbar level 5 (L_5_) and L_6_ vertebrae that had been dissected free of soft tissues were imaged in a μCT scanner (MS-09 Small Specimen Scanner; GE Healthcare, London, Canada). Images were reconstructed at 15-μm isotropic resolution (MicroViewABA 2.2; GE Healthcare). To measure qualities of the trabecular bone, the volume of interest was defined as the interior of each vertebral body, excluding the cortical bone. For measurements of the cortical bone, the volume of interest was defined as the cortical bone of the L_5_ vertebral body anterior to the neural arch, excluding the endplates. The inclusion of a standard phantom made of SB3 (a polymer with an X-ray attenuation across a wide range of voltages equivalent to bovine cortical bone, 1.18 g of hydroxyapatite/ml) during scanning was used for conversion of the CT attenuation values to BMD (mg/ml). The reconstructed grayscale images were thresholded to separate bone and marrow voxels. Using the thresholded data, tissue volume, bone volume fraction (bone volume/total volume), trabecular spacing, and mean cortical thickness were evaluated (MicroView ABA 2.2). A spinal segment including the L_5_ and L_6_ vertebrae from five animals per group were evaluated in this manner.

For histology and histomorphometry, isolated spines were dissected of soft tissue, fixed in 4% paraformaldehyde, decalcified, embedded in paraffin, sectioned, and stained with H&E (Histoserv, Germantown, MD, USA). Quantification of trabecular perimeter, length, and area was accomplished using software capable of measuring histomorphometric parameters (Bioquant Osteo II; Nashville, TN, USA). Data were gathered from five high-powered fields (×200)/specimen on at least five specimens per group.

### Immunohistochemistry

Sections were stained with antibodies against procollagen I (Developmental Studies Hybridoma Bank, Iowa City, IA, USA), Runx2 (MBL International, Woburn, MA, USA), or cathepsin K (Santa Cruz Biotechnology, Santa Cruz, CA, USA) to identify the presence of osteoblasts, osteoblast precursors, and osteoclasts in the bone, respectively. Sections were incubated overnight at 4°C with a mouse procollagen I antibody (clone SP1.D8; 1:50 dilution), a mouse Runx-2 antibody (clone 8G5; 1:50 dilution), or a goat cathepsin K antibody (1:100 dilution) in 2% BSA in PBS. Corresponding IgG antibodies were used as negative controls. Immunodetection was accomplished using a biotynilated link-streptavidin kit (DAKO) or the Vectastain universal Quick kit (Vector Laboratory, Burlingame, CA, USA) and visualized with a diaminobenzidine (DAB) chromogen detection system (DAKO) and Harris hematoxylin (PolyScientific, Bayshore, NY, USA) counterstain. Quantification of DAB^+^ cells was performed on five high-powered fields (×200)/specimen on at least five specimens per group (Bioquant Osteo II). The osteoblast (procollagen I–positive cell) and osteoclast (cathepsin K–positive cell) number was expressed as the total number of DAB cells and normalized to the total trabecular perimeter per high-powered field.

### Compression testing of L_6_ vertebrae

Intact lumbar level 6 (L_6_) vertebral bodies were dissected free of all soft tissues and tested to failure in compression.(19) Before testing, vertebral dimensions were determined by measuring the height along the cranial–caudal axis of the centrum, the width along the left–right axis, and the depth along the dorsal–ventral axis using calipers (±0.002 mm). Cross-sectional area was calculated from the length and width assuming an oval geometry.

Compression tests were performed between stainless steel platens on a servohydraulic testing system (Minibionix 858; MTS Systems, Minneapolis, MN, USA). A thin coating of cyanoacrylate adhesive was applied to the vertebral endplates to prevent slipping on the platens. An alignment pin attached to the lower platen was placed through the dorsal nerve tube to align and orient the vertebra vertically relative to the load. Compression was applied at 0.05 mm/s to a maximum load of 75 N. Ultimate (Fu) and yield (Fy) force, compressive stiffness (EA), displacement to failure (Du), and energy to failure were determined. After testing, vertebrae were defatted in acetone and dried at 60°C for 6 h to determine dry weight and then ashed at 600°C to determine mineral content.(20) Ash fraction (%) was calculated ash mass per dry mass × 100.

### Quantitative PCR

For quantitative PCR, mRNA was isolated from bone marrow flushed out from both tibias/mouse and resuspended to 1 ml TRIzol reagent (Invitrogen). RNA was extracted using RNeasy MinElute Cleanup Kit (Invitrogen). Expression levels of Runx-2, PTH/PTHrP receptor 1 (PPR), RANKL, and osteoprotegerin (OPG) in total bone marrow of AdShhN-treated or control animals (*n* = 3–5, all groups) were measured by TaqMan real-time RT-PCR using primers and probes from the manufacturer (Applied Biosystems, Foster City, CA, USA). The PCR reactions were run in an Applied Biosystems Sequence Detection System 7700. Relative expression levels were calculated using the ΔΔCt method (Applied Biosystems) with 18sRNA as the internal control and the average of AdNull samples as the calibrator.

### Colony-forming unit fibroblast, osteoblastogenesis, and osteoclastogenesis assays

To measure the number of mesenchymal stem cells, osteoblast precursors, and osteoclast precursors in the bone marrow, in vitro differentiation assays were performed using bone marrow cells harvested from the femurs of AdShhN-treated and control animals (*n* = 5) 18 days after vector administration. To measure the number of mesenchymal stem cells, triplicate aliquots of 5 × 10^5^ nucleated cells were plated on 35-mm dishes in Mesencult medium (StemCell Technologies, Vancouver, British Columbia, Canada), and colonies were counted by brightfield microscopy after 11 days.(21) To quantify the number of osteoblast progenitors in the bone marrow, triplicate aliquots were initially plated at 5 × 10^5^ cells/0.5 cm^2^ in mesenchymal stem cell sensitization media consisting of αMEM, 20% FBS (Invitrogen, Carlsbad, CA, USA), 1% penicillin-streptomycin (Gibco, Grand Island, NY, USA), and 55 μM 2-mercaptoethanol (Gibco). When the cells had grown to 70% confluency, the medium was changed to osteogenic medium consisting of αMEM, 20% FBS, 1% penicillin-streptomycin, 10 mM β-glycerophosphate (Lonza, Alendale, NJ, USA), 200 nM dexamethasone (Lonza), and 50 μg/ml ascorbate (Lonza). Cultures were grown for 14 days, and the presence of osteoblasts was confirmed by staining the cultures with Alizarin red-S. To quantify the extent of mineralization, an indicator of osteoblastogenesis, stained cultures were incubated in 10% cetylpyridinium chloride solution (Sigma, St. Louis, MO, USA) to release the Alizarin red-S dye, and dye concentration was measured at an absorbance wavelength of 570 nm. To measure the number of osteoclast precursors, triplicate aliquots of 3 × 10^5^ cells/ml were cultured in media containing αMEM, 10% FBS, 1% penicillin-streptomycin, and macrophage colony stimulation factor (M-CSF, 20 ng/ml; PeproTech, Rocky Hill, NJ, USA) for 24 h. Nonadherent cells were removed and replated at 2 × 10^5^ cells/0.5 cm^2^ in osteoclast differentiation medium consisting of αMEM, 10% FBS, 1% penicillin-streptomycin, M-CSF 20 ng/ml, and RANKL 100 ng/ml (R&D Systems, Minneapolis, MN, USA). Cultures were fixed on day 6 and stained with a leukocyte acid-phosphatase/TRACP kit (Sigma). Osteoclasts that were TRACP^+^ with more than three nuclei were counted in the entire culture at ×10 magnification.

### Osteoclast differentiation on stromal cell cultures

The capacity for bone marrow stromal cells (including mesenchymal and osteoblast progenitors) to support osteoclastogenesis was measured in co-cultures.(22) Briefly, stromal monolayers were established as described above in the osteoblastogenesis assay from triplicate cultures of bone marrow cells harvested from AdShhN-treated or control mice (*n* = 5). Untreated osteoclast progenitors were isolated from naive C57BL/6 mice by culture of whole bone marrow in media containing αMEM, 10% FBS, 1% penicillin-streptomycin, and M-CSF 20 ng/ml for 24 h. These osteoclast precursors were added to stromal cells cultures, and co-cultures were grown in stromal stimulation medium containing αMEM, 20% FBS, 1% penicillin-streptomycin, 20 nM PTH(1,34) (Bachem, Torrance, CA, USA), and 20 nM 1α,25-dihydroxyvitamin D_3_ (Sigma). To test the role of PTH receptor activation in co-cultures, triplicate wells were also supplemented with vitamin D_3_ alone or vitamin D_3_ and a PTHrP blocker, 20 nM PTH-RP(7-34) amide (Bachem). Osteoclast number was quantified on day 6 as described above.

## RESULTS

### Remodeling of vertebral trabecular bone in mice with increased serum levels of ShhN

**FIG. 1:**
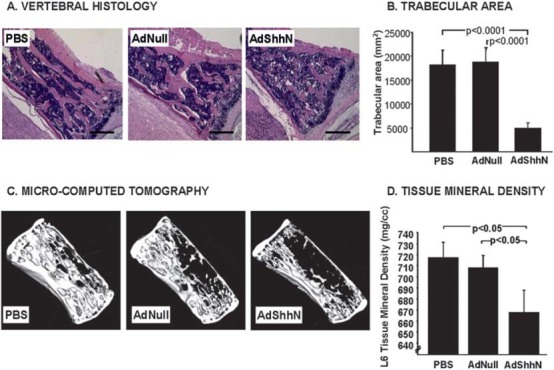
Remodeling of vertebral trabecular bone induced by elevated levels of Shh. Vertebrae from C57BL/6 mice were harvested 18 days after intravenous administration of adenovirus encoding a soluble form of Shh (AdShhN), a control adenovirus without a transgene (AdNull), or PBS. Microscopic morphology of mouse vertebrae were examined in histologic sections stained with H&E by brightfield microscopy or by μCT. (A) H&E images of vertebrae. PBS (left; AdNull (middle); and AdShhN (right). AdShhN-treated vertebrae show remodeling of trabecular architecture. Bar = 200 μm. (B) Total trabecular area in H&E-stained sagittal sections (*n* = 5 for all groups). (C) Reconstructed μCT image of a 370-μm midline sagittal section through the lumbar level 6 (L_6_) vertebra. (D) Tissue mineral density of L_6_ vertebrae (*n* = 5 for all groups). For B and D, data shown as mean ± SE.

As expected, serum Shh levels were elevated in AdShhN-treated animals, whose levels were 2.5 ± 1.7 ng/ml 18 days after vector administration. In contrast, serum levels of Shh in PBS and AdNull-treated controls was below the limit of detection. As shown by H&E-stained sections of the spine by light microscopy, vertebrae of AdShhN-treated mice showed a striking alteration in the trabecular architecture, characterized by an increased number of small trabeculae (Fig. 1A). Histomorphometric analysis showed that the total trabecular perimeter increased by 60% with AdShhN treatment (*p* < 0.0001 AdShhN versus both controls), indicating an increase in trabecular surface area. However, this was at the expense of trabecular area, which decreased by 70% (*p* < 0.0001, AdShhN versus both controls; Fig. 1B), suggesting a decrease in trabecular volume. Examination of the endosteal surfaces showed increased tortuosity. Consistent with these findings, μCT analysis showed that tissue mineral density was ∼7% lower in the trabeculae of Shh-treated L_5_ (*p* < 0.05) and L_6_ (*p* < 0.01) vertebrae compared with both controls (Figs. 1C and 1D; Table 1) and that bone volume fraction was decreased by ∼30% in L_5_ and 25% in L_6_ vertebrae of Shh-treated animals compared with control (*p* < 0.05, AdShhN versus both controls; Table 1). Changes in the average trabecular thickness were not detected by μCT. However, the average separation between trabeculae increased by ∼40% in L_5_ and 30% in L_6_ vertebrae of Shh-treated spines compared with control (*p* < 0.05 AdShhN versus both controls; Table 1). Evaluation of the cortical bone by μCT was also consistent with endosteal remodeling showing that cortical thickness decreased by ∼15% and that bone volume fraction was decreased by ∼10% (*p* < 0.05, AdShhN versus PBS; *p* < 0.01, AdShhN versus AdNull for both parameters; Table 2) but that tissue mineral density was not affected (*p* > 0.2, AdShhN versus both controls; Table 2).

**Table 1:** Decreased Trabecular BMD in L_5_ and L_6_ AdShhN Vertebral Bodies After Treatment With AdShhN^*^

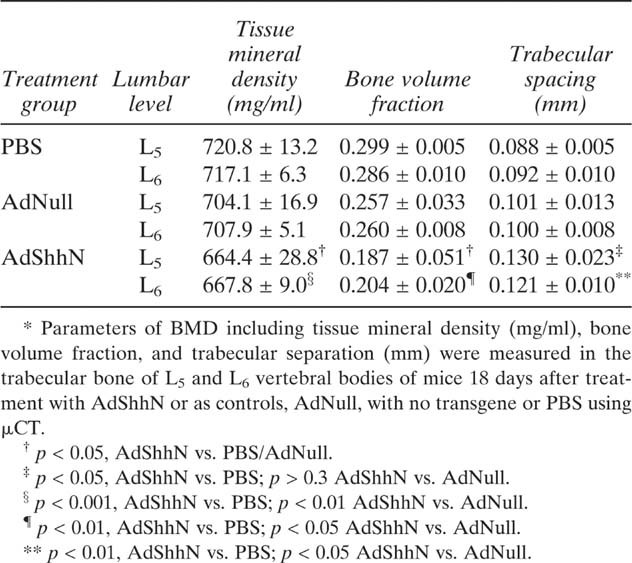

**Table 2:** Decreased Cortical Bone Thickness in L_5_ Vertebral Bodies After Treatment With AdShhN^*^

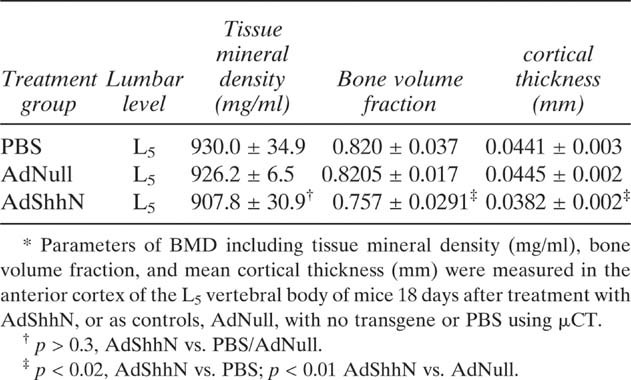

### Decreased compressive properties in AdShhN-treated vertebrae

**FIG. 2:**
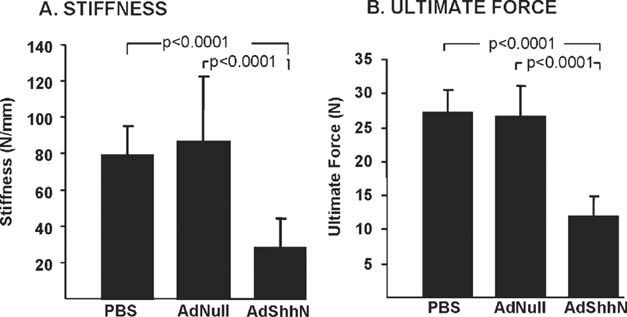
Decreased biomechanical properties of AdShhN-treated vertebrae. Isolated lumbar level 6 (L_6_) vertebra (*n* = 10 vertebrae/group) were oriented axially relative to the load and compressed at 0.05 mm/s to a maximum load of 75 N and then ashed after the mechanical testing. (A) Compressive stiffness. (B) Ultimate force. Data shown as mean ± SD.

**FIG. 3:**
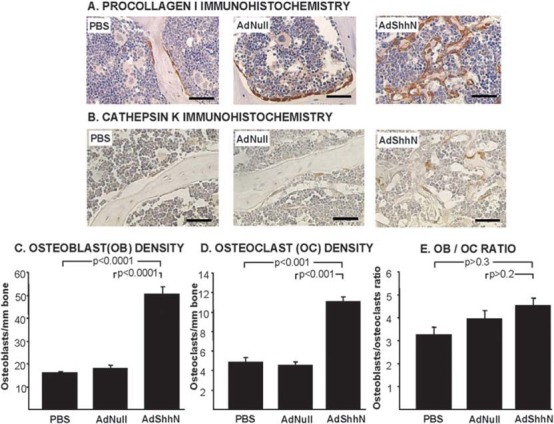
Osteoblasts and osteoclasts increase in vertebrae 18 days after AdShhN treatment. (A) Immunohistochemical staining for procollagen I, a cytoplasmic marker of osteoblasts: PBS (left); AdNull (middle); and AdShhN (right). Brown indicates positive staining. Bar = 40 μm. (B) Immunohistochemical staining for cathepin K, a marker of active osteoclasts: PBS (left); AdNull (middle); and AdShhN (right). Bar = 40 μm. (C–E) Quantification of osteoblast and osteoclast numbers (*n* = 5 for all groups). (C) Osteoblasts/mm of trabecular area. (D) Osteoclasts/mm of trabecular area. (E) Osteoblast/osteoclast ratio. For C–E, data shown as mean ± SE.

To determine the functional effects of the changes in trabecular architecture seen by histology and μCT, the mechanical properties of L_6_ vertebrae were measured in compression. Compressive stiffness was significantly reduced in AdShhN-treated vertebrae (*p* < 0.0001 AdShhN versus both controls) and was nearly 3-fold lower on day 18 (Fig. 2A) and nearly 4-fold lower on day 30 (Table 3) compared with both control groups. Compressive failure strength was also significantly reduced in AdShhN-treated vertebrae relative to the control groups (*p* < 0.0001), by >2-fold at 18 days (Fig. 2B) and 3-fold on day 30 (Table 3), compared with both control groups. Yield strength was reduced with AdShhN-treatment similar to the failure strength changes at both time points. Energy and displacement to failure were not affected by treatment.

**Table 3:** Diminished Biomechanical Properties of L_6_ Vertebra After Treatment With AdShhN^*^

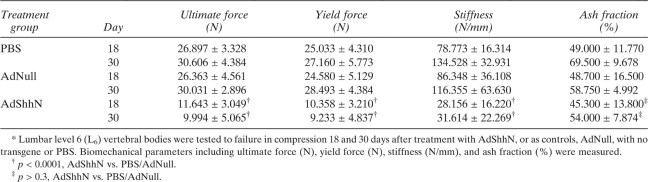

Vertebral height and depth were both significantly decreased after AdShhN treatment relative to both control groups. The mineral content and ash fraction were not significantly affected by AdShhN treatment (Table 3).

### Coupled increases in osteoblasts and osteoclasts in vertebrae after AdShhN treatment

To begin to examine the mechanism underlying the changes in the trabecular architecture, the presence of activated osteoblasts and osteoclasts was determined by immunohistochemistry. As expected, an ∼2-fold increase in the number of osteoblasts, positive for procollagen I, was detected in AdShhN-treated vertebrae compared with controls (*p* < 0.0001, all comparisons; Figs. 3A and 3C). At the same time, an increase in the number of active osteoclasts, positive for cathepsin K, was also detected in AdShhN (Fig. 3B). Because the magnitude of the increase in osteoclasts was similar to that of osteoblasts (*p* < 0.001, all comparisons; Fig. 3D), the ratio of osteoblasts to osteoclasts was not different between AdShhN and control animals (*p* > 0.2 versus AdNull, *p* > 0.3 versus PBS; Fig. 3E).

### Primary increase in osteoblast precursors and stimulation of osteoblastogenesis after AdShhN treatment

**FIG. 4:**
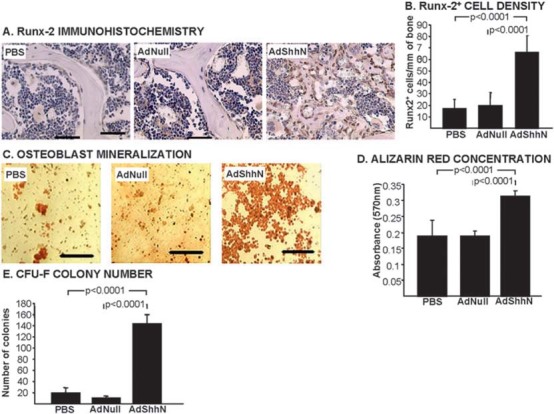
Immature osteoblasts, osteoblast precursors, and mesenchymal stem cells increase with AdShhN treatment. (A) Vertebrae stained for runt-related transcription factor-2 (Runx-2), a marker of early osteoblasts 18 days after treatment: PBS (left); AdNull (middle); and AdShhN (right). Brown indicates positive staining. Bar = 40 μm. (B) Quantification of Runx-2–positive cells/mm of trabecular surface area (*n* = 5 for all groups). (C–E) Osteoblastogenesis and CFU-F assays were performed using cultured bone marrow cells from AdShhN and control mice. Alizarin red-S staining for mineralization after 3 wk of culture in osteogenic media: PBS (left); AdNull (middle); and AdShhN (right). Bar = 80 μm. (D) Alizarin red-S concentration. (E) CFU-F number per well after 14 days. For B, D, and E, data shown as mean ± SE.

To study further whether the primary effect of Shh treatment was on the osteoblast precursors, osteoblastogenesis was examined in vivo and in vitro. A 2.5-fold increase in the number of Runx2-positive cells in the vertebrae of AdShhN-treated animals (*p* < 0.0001, all comparisons; Figs. 4A and 4B) showed that, in addition to the mature, active procollagen I–positive osteoblasts (Fig. 3A), osteoblast precursors were also increased. To confirm these findings, bone marrow cells were cultured, and in vitro assays were performed to measure the presence of osteoblast precursors and of mesenchymal stem cells. A 65% increase in the number of mineralizing nodules in AdShhN-treated bone marrow cells confirmed an increase in the number of osteoblast precursors in those cultures (*p* < 0.0001; Figs. 4C and 4D). Similarly, the colony-forming unit fibroblast (CFU-F) assay confirmed a 7-fold increase in the number of mesenchymal stem cells in AdShhN-treated bone marrow (*p* < 0.0001 all comparisons; Fig. 4E).

### No increase in the number of osteoclast precursors but enhanced osteoclastogenic potential of stromal cells after AdShhN treatment

**FIG. 5:**
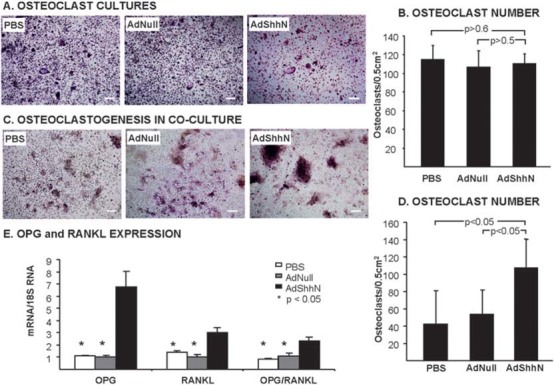
No change in the number of pre-osteoclasts, but enhanced osteoclastogenic potential of bone marrow stromal cells after AdShhN treatment. (A and B) Osteoclast precursors were harvested from bone marrow 18 days after AdShhN treatment and cultured with osteoclast-inducing medium for 6 days. (A) Examples of osteoclast cultures stained with TRACP (PBS, left; AdNull, middle; and AdShhN, right; bar = 100 μm) and (B) quantification of TRACP^+^ osteoclasts. Total number of TRACP^+^ cells with greater than three nuclei per cell were counted (*n* = 5, all groups). (C and D) Osteoclastogenesis was also measured in precursors harvested from naive mice and co-cultured on monolayers of bone marrow stroma established from AdShhN-treated or control mice. Co-cultures were supplemented with dihydroxyvitamin D_3_ and PTH. (C) Examples of co-cultures stained with TRACP (PBS, left; AdNull, middle; and AdShhN, right; bar = 100 μm) and (D) quantification of osteoclasts in co-cultures. For B and D, data shown as mean ± SE. (E) Expression levels of OPG and RANKL expression measured in RNA extracted from femoral bone marrow cells 18 days after vector administration and quantified by TaqMan real-time PCR (*n* = 5, all groups). Data shown as mean ± SE.

To study whether Shh also had a primary effect on osteoclast precursors, in vitro osteoclastogenesis assays were performed. In contrast to the osteoblastogenesis assays, osteoclastogenesis assays failed to show any differences between treatment groups (*p* > 0.5 versus AdNull, *p* > 0.6 versus PBS; Figs. 5A and 5B), indicating that, whereas Shh treatment did increase the number of osteoblast precursors, it had not increased the number of osteoclast precursors. To test whether the increase in osteoclasts in vivo was a result of Shh-induced changes to the bone marrow stroma, co-cultures assays were performed. When bone marrow–derived osteoclast precursors isolated from naive, untreated animals were co-cultured on stromal cells harvested from AdShhN-treated animals, a 3-fold greater number of osteoclasts were formed compared with co-culture on stroma from AdNull- or PBS-treated controls (*p* > 0.05, all comparisons; Figs. 5C and 5D; Supplemental Figs. S1A and S1B). Together, these data suggest that the effect on osteoclasts is secondary and is caused by enhanced ability of AdShhN-treated stroma to support osteoclastogenesis.

Because osteoclastogenesis is regulated by OPG and RANKL molecules secreted by osteoblast precursors/stromal cells,(3,23,24) we determined the levels of these molecules. As expected, RANKL gene expression was elevated 1.5-fold in AdShhN-treated stroma (*p* < 0.05 versus AdNull or PBS; Fig. 5E). A 5-fold increase in OPG gene expression with Shh treatment was also in agreement with our observation of increased osteoblasts (*p* < 0.05 versus AdNull or PBS; Fig. 5E). A 1.4-fold increase in the OPG:RANKL ratio in Shh-treated marrow (*p* < 0.05; Fig. 5E) was somewhat surprising, because this would suggest a bone-forming state rather than bone resorption.

### Enhanced osteoclastogenic potential of AdShhN-treated stroma is associated with increased activation of the PTH/PTHrP pathway

**FIG. 6:**
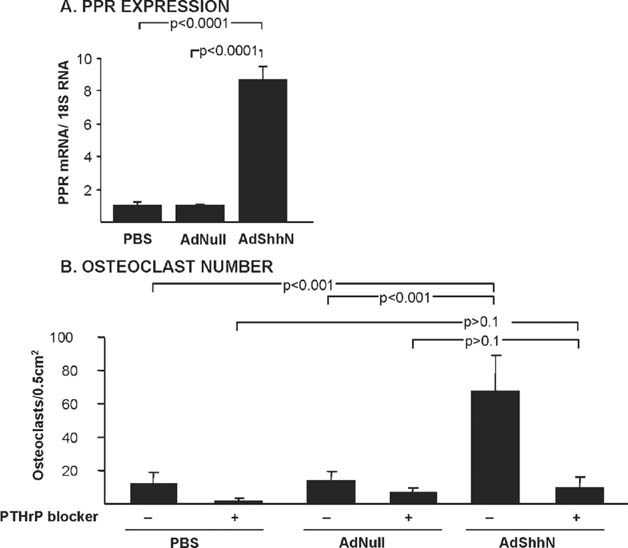
Enhanced osteoclastogenesis is associated with increased expression and activation of the PTH/PTHrP pathway. (A) Expression level of PPR measured in RNA extracted from femoral bone marrow cells 18 days after vector administration and quantified by TaqMan real-time PCR (*n* = 5, all groups). Data shown as mean ± SE. (B) Quantification of osteoclasts in co-cultures of marrow cells from animals treated with AdShhN or control and untreated osteoclast precursors cultured with (+) or without (−) PTHrP blocker. Cells were stained with TRACP on day 6 of culture and counted under brightfield microscopy (*n* = 5, all groups).

Because hedgehog signaling can have downstream effects on PTH/PTHrP signaling,(8) and because PTH and PTHrP are important for the growth and development of both osteoblasts and osteoclasts,(2,3,11,25,26) the role of PPR activation in AdShhN-induced increase in osteoclastogenesis was studied. When PTH was removed from the osteoclastogenesis media, and co-cultures stimulated with vitamin D_3_ alone, AdShhN stroma still had a 4-fold greater capacity to support osteoclastogenesis versus control stroma (*p* < 0.001, all comparisons; Fig. 6A). Addition of a PTHrP analog that blocks PPR activation abolished this effect, confirming that activation of the PTH/PTHrP pathway was responsible for the AdShhN-induced effect (Fig. 6A; Supplemental Fig. S1C). Whereas PTHrP expression levels were below the level detectable by quantitative real-time PCR analysis, analysis of fresh bone marrow showed that PPR gene expression levels were increased by >4-fold in AdShhN-treated animals (*p* < 0.0001 all comparisons; Fig. 6B), further supporting the role of PPR receptor activation as the mechanism for increased osteoclastogenic capacity in the AdShhN stroma.

## DISCUSSION

To study the effects of Shh on osteoprogenitors and bone formation in vivo, we overexpressed Shh in adult mice using an adenoviral vector expressing the N-terminal form of Shh. Our results showed that a systemic rise in serum Shh levels increases the numbers of mesenchymal stem cells, osteoblast precursors, and osteoblasts in the bone marrow but results in the remodeling of trabecular bone and reduction in bone strength caused by a secondary increase in osteoclasts. The enhanced osteoclastogenesis is induced by the stromal microenvironment and is mediated in part by the PTH/PTHrP pathway, likely because of upregulation of PPR. We conclude that Shh stimulates osteoblastogenesis in vivo, which leads to increased remodeling of trabecular bone.

### Osteoprogenitors and osteoclastogenesis

Remodeling is a coordinated process of bone resorption and formation that requires the balanced interaction between osteoblastogenesis and osteoclastogenesis and is essential for maintaining normal BMD. The osteopenic phenotype observed in our study is nearly identical to that very recently reported by Mak et al.(27) and Ohba et al.(28) in transgenic mice with upregulated hedgehog signaling through deletion of one copy of the hedgehog receptor, Patched1, and it highlights, as do their studies, the importance of the osteoblast progenitor in maintaining BMD. Our finding of a remarkably rapid and complete remodeling of bone resulting in highly significant changes in the appearance and mechanical function of the vertebrae within 18 days attests to the importance of this pathway, not only in development, as shown in the transgenic models, but in postnatal bone. Furthermore, our studies provide a compliment to that of Ohba et al.,(28) who reported an increase in BMD in postnatal mice through pharmacologic blockade of hedgehog signaling.

Previous studies have reported on the stimulatory effects of Shh on osteoblastogenesis and mesenchymal stem cells.(7–9,14,29–31) Our study, along with those using Patched1 transgenics show, however, that when stimulated in situ by Shh, the osteoprogenitors (including marrow stromal cells and osteoblast precursors) support osteoclastogenesis rather than inducing bone formation.(27,28) As expected based on the increased osteoclastogenic capacity of Shh-treated stroma and based on the increase in the numbers of osteoprogenitor cells, we detected increased expression of RANKL, a requisite molecule for osteoclast maturation and survival that is preferentially expressed on less mature osteoblasts and their precursors.(32,33) However, we also detected an increase in OPG expression, which, while consistent with the increase in type I collagen–positive osteoblasts induced by Shh treatment, resulted in a surprising elevation of the OPG:RANKL ratio that usually declines in bone resorptive states.(2) Several factors may have contributed to this unexpected finding.

One possibility is that factors other than RANKL are driving bone resorption in our model. For example, IL-6 has been reported to increase osteoclast activity with or without enhancing the RANK-RANKL system,(34) and studying its expression in our model in the future might be revealing. Another example is the potent (non-RANKL mediated) stimulation of osteoclast activity by immature bone matrix. Such matrix–osteoclast interactions are theorized to be the mechanism of decreased BMD in the Runx2 overexpressing transgenic mouse.(35–37) Further study of the matrix quality in our model could show whether such changes also occurred with Shh treatment. Previous studies reporting cross-regulation of hedgehog and Runx2 activities(28,38,39) support the possibility that the increase in Runx2-positive osteoblast precursors in our study may reflect not only a Shh-enhanced osteoblastogenesis, but a direct effect of Shh on Runx2 upregulation. To study non–RANKL-mediated factors that may stimulate osteoclast activity in our model is of interest for future investigations.

Anatomical variation is another consideration. In some studies of osteoporosis, correlations between BMD and the OPG:RANKL ratio were found in some anatomic locations but not others.(40,41) In this light, measuring OPG:RANKL in the vertebrae instead of the femurs may have shown an tighter correlation with BMD in the vertebrae. Incidentally, our studies on hematopoiesis in our model showed the appearance of small bone islands in the femoral diaphysis, even as the femoral metaphysic underwent a similar decrease in BMD as the trabecular bone of the vertebrae (M. Kiuru, C. Hidaka, R. Hubner, J. Solomon, A. Krause, P. Leopold, R. Crystal, unpublished data, 2009). It is possible that increased bone formation in the femoral diaphysis may have resulted in the increased OPG:RANKL ratio.

Additionally, it is worth noting that a decrease in the OPG:RANKL ratio is not always found in every instance of decreased BMD or trabecular thinning. For example, Fazzalari et al.,(42) found that, in osteoarthritis, the OPG:RANKL ratio relationship was not predictive of parameters of trabecular bone thinning. Expression of RANKL itself, however, did correlate (inversely) with BMD. This observation may suggest that the absolute rise in RANKL (and its primary pro-osteoclastogenic properties) may be more important in predicting BMD than OPG, which acts by suppressing RANKL.

### PTH/PTHrP pathway activation

In addition to the increased expression of RANKL, we found an important role for the PTH/PTHrP pathway on osteoclastogenesis in the context of Shh treatment. Our findings are similar to those reported by Mak et al.,(27) showing upregulation of PTHrP expression. Whereas we did not detect a rise in PTHrP expression, we did find increased expression of the receptor PPR and also found that blockade of the receptor could inhibit the increased osteoclastogenic capacity of Shh-treated stoma. One possibility for the lack of any change in PTHrP expression levels in our study is the short half-life and instability of PTHrP mRNA.(43,44) Whereas PTHrP expression levels may have remained elevated in a transgenic mouse model, it may have been more transient in our Ad-mediated transient gene transfer study.

Consistent with our observations regarding the PTH/PTHrP pathway, the increased number of osteoblasts and osteoclasts as well as the appearance of small trabeculae in Shh-treated mice has also been observed in transgenic mice with constitutively activated PPR.(45) That, in contrast to our model, the PPR transgenic mice also showed a substantial increase in trabecular bone volume may be caused by developmental effects that would not be expected in our postnatal model.

### Osteoprogenitors and the control of BMD

In showing the importance of osteoprogenitors in bone remodeling, our data support the current clinical practice of adding bone marrow cells to bone graft and suggest furthermore that marrow cells activated with Shh or otherwise enriched in early precursors may further enhance graft remodeling and possibly graft incorporation. More important, our model adds to the growing body of recent literature supporting the central role of the hedgehog pathway and of osteoblast precursors in maintaining BMD in postnatal animals. Furthermore, our study supports the counter-intuitive approach of judiciously suppressing osteoblastogenesis for producing increased BMD. In addition to the study of Ohba et al.(28) using hedgehog inhibition to increase BMD, other reports of transgenic mice with impaired osteoblastogenesis (through expression of dominant negative Runx2 or haplo-insufficiency or dickopf-1, for example) that show increased BMD support such a contention.(46–48)
